# MEM&SO protocol: understanding the determinants of social learning in neurodegenerative diseases

**DOI:** 10.1186/s40359-024-01791-w

**Published:** 2024-05-28

**Authors:** Pauline Saliou, Julien Chavant, Serge Belliard, Catherine Merck, Vincent de La Sayette, David Wallon, Olivier Martinaud, Francis Eustache, Mickaël Laisney

**Affiliations:** 1 Inserm, U1077, EPHE, UNICAEN, Normandie Université, PSL Université Paris, CHU de Caen, GIP Cyceron, Neuropsychologie et Imagerie de la Mémoire Humaine (NIMH), Caen, 14000 France; 2 Département de Neurologie, CHU Pontchaillou, Rennes, France; 3 Univ Rouen Normandie, Normandie Univ, Inserm U1245 and CHU Rouen, Department of Neurology and CNRMAJ, Rouen, F- 76000 France

**Keywords:** Alzheimer disease, Aphasia, primary progressive, Social learning, Personality, Social interaction.

## Abstract

**Background:**

People with neurodegenerative diseases may have difficulty learning new information, owing to their cognitive impairments. Teaching them techniques for learning in social contexts could alleviate this difficulty. The present study will examine the performances of patients with Alzheimer’s disease and patients with the semantic variant of primary progressive aphasia on a memory test administered in three social contexts. The protocol will make it possible to identify determinants of social interactions, social abilities, cognition, and personality that can explain the potentially beneficial effect of social context on learning in these patients.

**Methods:**

Thirty dyads (patient with primary memory impairment who meets criteria for Alzheimer’s disease paired with caregiver), 16 dyads (patient meeting criteria for semantic variant of primary progressive aphasia paired with caregiver), and 46 dyads (healthy controls with no cognitive complaints) will be recruited. A nonverbal memory test (social memory task) will be administered to each dyad in three different social contexts (presence-only, observation, collaboration). Patients and healthy controls will also undergo a neuropsychological assessment to measure social (interactions and abilities), cognitive and personality aspects. Patients will be compared with controls on differential social scores calculated between the presence-only and collaboration contexts, and between the presence-only and observation contexts. A multiple comparative case study will be conducted to identify social, cognitive and personality variables that potentially explain the differential scores in the collaboration and observation contexts.

**Discussion:**

For the first time, memory will be assessed in patients with Alzheimer’s disease and patients with the semantic variant of primary progressive aphasia in three different contexts (presence-only, observation, collaboration). The multiple comparative case study will make it possible to identify the determinants of memory performance in the social context, in order to create the most beneficial learning context for individual patients, according to their profile.

**Trial registration:**

This study was approved by the Ile de France XI institutional review board (2022-A00198-35), and registered on ClinicalTrials.gov (no. NCT05800028), on April 27, 2023.

## Background

Neurodegenerative diseases place patients in situations of dependence in which they have to interact with people who are often new to them and adapt to new living environments. These life changes involve learning and memorizing new information, which is particularly difficult for people with cognitive disorders, such as memory impairment in Alzheimer’s disease (AD) and language deficits in the semantic variant of primary progressive aphasia (svPPA). The amnesic form of AD, characterized by an initial and predominant memory disorder, is the commonest and most typical form of the disease [1]. Severe deficits in learning new information are a core feature of AD, although deficits in storage and retrieval cannot be excluded [2, 3]. A progressive loss of semantic knowledge is observed in svPPA [4]. The latter is characterized by an initial and predominant impairment of language, with poor verbal comprehension and profound word-finding difficulties that interfere with daily activities and include the impairment of confrontation naming and single-word comprehension. Current and proposed classifications suggest that svPPA and semantic behavioural variant frontotemporal dementia [5] are both early forms of semantic dementia. The latter is characterized by a gradual loss of semantic knowledge evidenced by difficulty finding words and identification deficits for objects and/or persons, and impaired word comprehension [6, 7].

Different learning techniques have been developed to try to compensate for the learning and memory deficits of patients with AD, or improve the naming and use of objects for patients with svPPA, in order to maintain their autonomy. *Errorless learning* acts on the encoding phase, the aim being to avoid learning incorrect information [8–11]. The *vanishing cues method* facilitates the retrieval of information by providing cues that gradually disappear [12, 13]. The purpose of *space retrieval* is to improve long-term retention through the repetition of learning with increasingly long intervals between the encoding of information and its retrieval [14, 15]. In patients with svPPA, the semantic features of concepts are enriched [16, 17], and object use is relearned [18].

All these techniques involve the creation of materials for each learning event and require numerous repetitions, but they do not always have observable long-term effects, and generalization to other types of material is not always effective or tested [19]. For patients with PPA, the most beneficial strategy is functional communication, where communication strategies that are already used by the patient are practised with a communication partner [20]. However, this and other similar techniques need to be performed with others, and cannot be practised alone.

Numerous social psychology studies among healthy individuals have shown that the *presence of others* can have, either a positive effect on cognitive performance (i.e., *social facilitation*) or a negative effect, (i.e., *social inhibition*). In memory tasks, both social facilitation and inhibition depend, on memory consolidation [21]: if learning is not consolidated, the presence of others during encoding has a negative impact on learning speed. Although there have been relatively few studies of memory tasks, results suggest that the social effect differs according to recall interval. While short-term recall (2 min) is impaired when individuals are observed during encoding [22], their performance improves as the time between encoding and recall increases (i.e., 15 min [23], and 45 min [22]). By contrast, when individuals are observed during a neuropsychological assessment, their cognitive performance decreases (i.e., social inhibition), with attention and memory tasks (immediate and delayed recall) being most negatively affected [24]. The nature of the social effect (i.e., facilitation vs. inhibition) may also depend on the real or perceived difficulty of the task [25], as well as on the individual’s assessment of the other person [26] or of the context in general. In other words, depending on the individual’s personality traits, assessment of the context, and feelings towards the task, learning in the presence of others may generate pressure through apprehension of the task and the fact of having to tackle its difficulties alone.

This pressure may be alleviated and cognitive load reduced in a context where individuals can observe someone else carrying out the task, insofar as they no longer have to tackle it themselves. *Observational learning* (i.e., receiving information, then using it) builds habits and improves observers’ skills [27]. However, although observational learning is advantageous because it avoids the cost of trial-and-error learning, individuals need to be selective, only using the relevant information that comes from learning from others [28, 29]. Observational learning of a motor sequence seems to be efficient in patients with AD [30].

Collaborative learning, through partners’ co-construction of ideas within what can be regarded as a particular memory group [31], is more effective than learning on one’s own [32]. *Collaborative learning* is defined as interaction between peers with the aim of jointly performing a task, and meets three criteria: communication, reciprocity, and common goal [33]. Collaboration is said to be beneficial for learning [34], and contributes to participants’ social, emotional and psychological wellbeing (e.g., collaborative learning at university [35]). However, collaboration may have some disadvantages, such as the possible dilution of motivation, decreased productivity if there are uneven contributions from members, and possible encoding of errors produced by collaborators and played back in subsequent recall [32]. Collaborative learning outperforms learning alone, both among older people undergoing the normal age-related deterioration in memory [36] and among patients with AD [37, 38]. The social dimension, including common idea building and conversational skills, therefore seems to constitute a favourable learning environment. Using a collaborative trial in which patients self-generated labels with a familiar partner, Duff and colleagues [37] showed that patients with AD can perform comparably to healthy individuals. Beyond their ability to build a common representation with their partner, patients with AD are capable of knowing that other people do not share this representation [39]. However, the mechanisms behind this effect are still poorly understood, given that these patients have impaired theory of mind (ToM; i.e., ability to attribute mental states to others) [40], an essential skill for acting appropriately in social exchanges. The presence of a social context may help to compensate for patients’ apparent difficulty constructing representations of others’ mental states [41]. Several neurological diseases are characterized by ToM disorders [42]. In semantic dementia, for instance, both cognitive and affective forms of ToM are affected [43], mainly as a result of right anterior temporal atrophy in the early stage of disease [5] or after bilateralization. Atrophy of the right anterior temporal lobe is correlated with impaired emotion recognition and person identification [5, 44–46]. Some patients may exhibit egocentric behaviour [47], and respond with their own personal preferences when judging the preferences of others [42]. These social behavioural disorders can be attributed to the ToM impairment reported in these patients [43], as well as to the impairment of semantic knowledge about social norms [42].

The main objective of the present study will be to assess whether social context (collaboration or observation) improves the memory performances of patients with a neurodegenerative disease who exhibit social cognition, memory or language disorders. A secondary objective will be to identify social (interactions and abilities), cognitive and personality variables that could explain differences in patients’ performances between collaborative and observation contexts.

To this end, a nonverbal memory test (social memory task) will for the first time be administered in three different social contexts (presence-only, observation, collaboration), to patients with AD, patients with svPPA, and healthy older people with no cognitive disorders. Participants will also undergo a neuropsychological assessment probing social (interactions and abilities), cognitive and personality aspects.

A multiple comparative case study will be conducted [48–50] to identify variables (social interactions and social abilities, cognitive skills, and personality traits) that could explain better performances in a collaboration or observation context than in a presence-only context.

## Methods

### Participants

We will recruit 184 participants in order to create 92 dyads: 30 dyads in which a person who meets the criteria for AD with primary memory impairment [1] is paired with a caregiver, 16 dyads in which a person who meets the criteria for svPPA [4] is paired with a caregiver, and 46 pairs of healthy controls with no cognitive complaints (HC).

The dyad partners must have socialized together for at least 2 h per week for at least 5 years. Patients and HC must be aged 50–85 years. Patients’ caregivers and HC must have no cognitive complaints and a Montreal Cognitive Assessment (MoCA) [51] score > 25.

Exclusion criteria for all participants will be (1) concurrent participation in a therapeutic drug trial, (2) prior neurological disorders (stroke, epilepsy, head injury with loss of consciousness lasting more than 1 h), (3) chronic alcoholism or drug addiction, (4) a clinically severe major psychiatric disorder within the previous 10 years, and (5) use of psychotropic medication.

The size of the patient groups was determined according to the feasibility of including this type of patient, based on studies carried out for more than 15 years in our research unit [43, 52, 53]. The size of the HC group was determined in relation to the size of the patient groups. To ensure that HC are not cognitively impaired, a quick cognitive test will be performed at the beginning of the psychological interview. Individuals who perform below the normal range will be excluded from the study and replaced.

### Social memory task

**Fig. 1:**
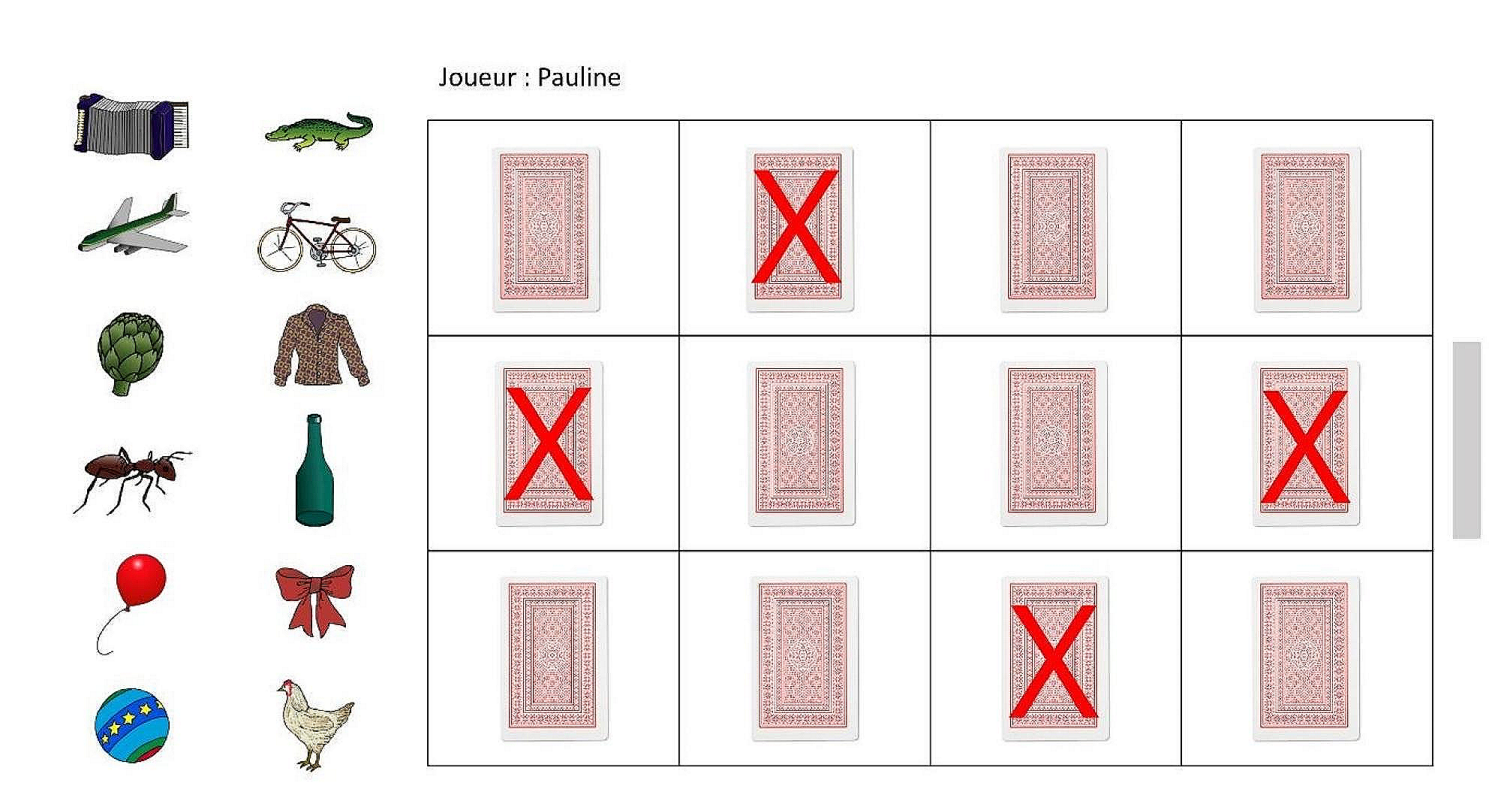
Screenshot showing learning phase of social memory task, displayed on touchscreen of computer

The social memory task takes the form of a game, and has been specially designed to be feasible in different social contexts and adapted to patients with cognitive disorders. The game consists in constructing 12 pairs of pictures by selecting a picture in a draw pile (always visible) and finding the same picture in a 12-box grid (hidden face; see Fig. 1). Participants (here, either patients or HC) are instructed to memorize the position of that match in the grid, as this will be tested later. A recall phase takes place 20 min after this learning phase. The game will be repeated in each of the three social contexts (presence-only, observation, collaboration; see “Procedure for social memory task” subsection below), with three different sets of pictures.

This test will be displayed on the touchscreen of a computer with a detachable keyboard. The 12.3-inch touchscreen will lie flat on the table for the duration of the test. The material used for this task was taken from Snodgrass and Vanderwart’s set of object pictures [54]. These images were improved with colour and texture [55], and ranked according to familiarity and image agreement. We selected the first 72 pictures, and randomly assigned them to one of the three contexts (i.e., 24 pictures per context). There were therefore 12 target pictures and 12 distractor pictures for each context. This task comes in two phases: picture location learning, and location recall.

#### Learning phase

Participants will have to match each of the 12 target pictures in the pile (lefthand side of screen) with an identical one hidden in the grid. In order to encourage them to explore the whole grid and avoid making the test too simple, for each trial, four boxes will be blocked: the box identified in the previous trial, and three others at random. When a pair is found, the relevant picture on the lefthand side of the screen will be marked with a green tick, and the participant will no longer be able to click on it. The learning phase will end when all the pairs have been constructed. To make the social memory task appear more like a game, participants will have to match each pair before the cursor on the righthand side of the screen is lowered. To avoid setting participants up for failure, the cursor will only be lowered after a pair is found, and not after each trial.

#### Recall phase

The recall phase will take place after an interference task (completion of a questionnaire), and will consist of picture recognition and grid location recall. For the picture recognition, all 24 pictures (i.e., 12 targets and 12 distractors) will be displayed one by one in random order. For each picture, participants will have to say whether or not they saw the picture in the learning phase. For the location recall, the 12 target pictures will be displayed one by one, and participants will have to place each one in the box it occupied in the learning phase.

#### Procedure for social memory task

The learning phase will take place in three social contexts: (1) *presence-only* context, where the participant performs the task alone, with the dyadic partner present in the same room but behind a screen; (2) *observation* context, where the participant observes the dyadic partner performing the task; and (3) *collaboration* context, where the participant and the dyadic partner perform the task together. Each dyad will perform the task in all three contexts. The order of the three contexts will be randomly counterbalanced across the dyads.

**Fig. 2:**
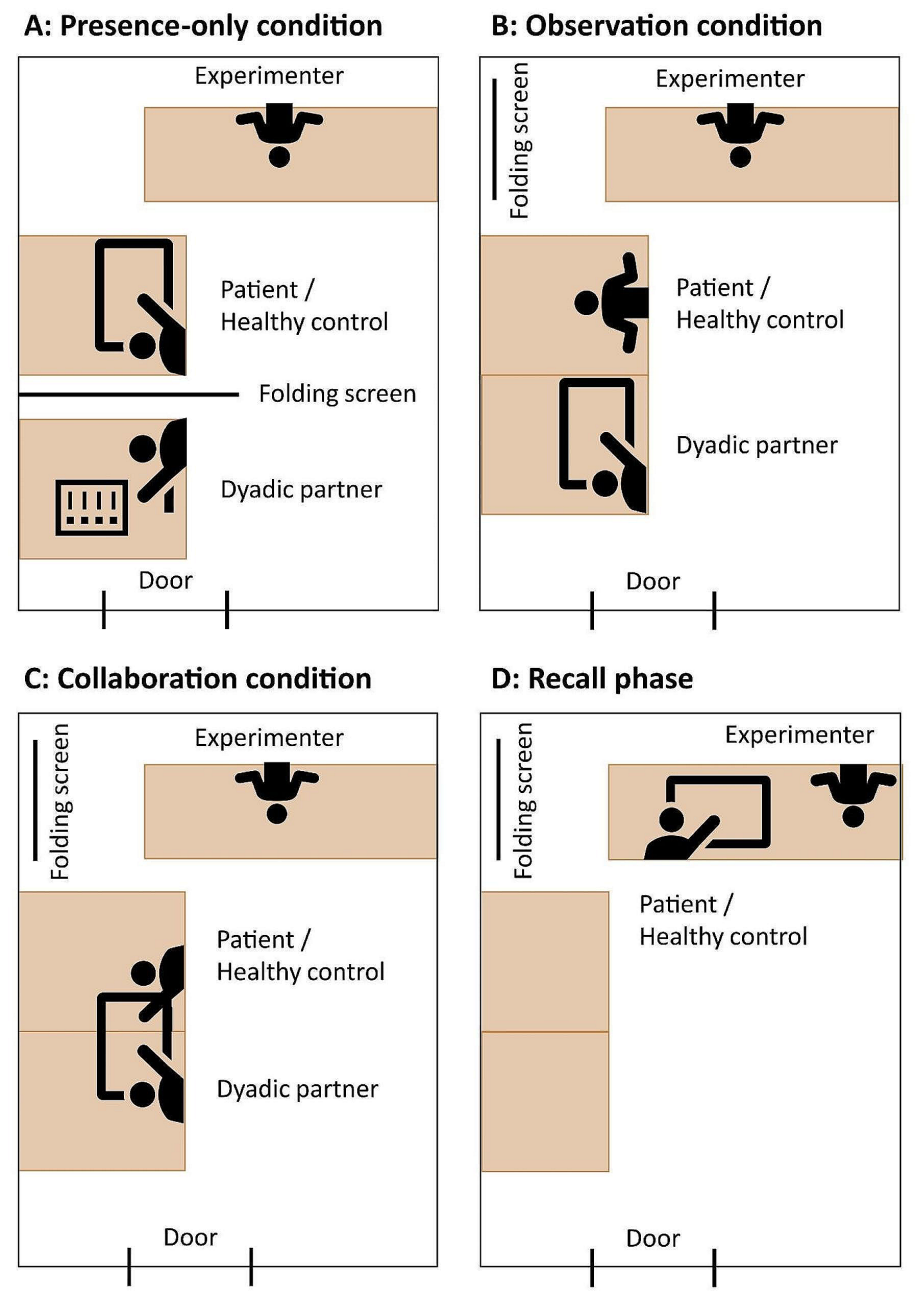
Layout of experiment room in (**A**) presence-only condition, (**B**) observation condition, (**C**) collaboration condition, and (**D**) recall phase

During the learning phase, regardless of the context (presence-only, observation, or collaboration), the participant, dyadic partner, and experimenter will all be present in the room (see Fig. 2 for room layout). The tables of the two members of the dyad will be placed side by side facing a wall, and the experimenter’s desk will be perpendicular to these two tables, more than 1 m away. In the presence-only context, the touchscreen will be placed between the participant’s table and the dyadic partner’s table. The dyadic partner will complete a questionnaire during this learning phase. In the observation and collaboration contexts, the dyadic members’ tables will be fixed together, and the touchscreen will be placed in the middle.

During in the recall phase, only the participant and the experimenter will be present in the room. The participant will be seated at the same table as the experimenter.

#### Outcome of social memory task

For the learning phase, and for each social context, several scores will be collected: the number of trials performed before all the pairs of pictures are found, the number of clicks on each picture on the lefthand side of the screen, and on each picture in the grid. We will examine whether these scores are related to delayed recall performance, to establish whether a picture is remembered better and more accurately located if it has been seen several times.

For the picture recognition in the recall phase, we will record the numbers of correct responses (i.e., saying “yes” when the picture has previously been seen, and saying “no” when the picture has not been seen before) for each social context.

Finally, for the location recall, we will calculate an overall score for each social context. If a picture is correctly located in the grid, 1 point will be awarded, and if it is located in an adjacent box, 0.5 point will be awarded. If not, 0 point will be awarded.

We will assess whether social context influences the picture recognition and location recall scores, and use these scores to identify factors that could explain potential differences in recognition performance between social learning contexts.

#### Debriefing

To clarify the psychological mechanisms associated with performance in different social contexts, a debriefing phase will take place at the end of each learning session. Using Likert-type scales, participants will rate their confidence in the quality of the learning achieved, their pleasure and satisfaction during the task, their anxiety during the learning process, the effort put into learning, the attention paid to the task, and perceived task difficulty.

### Neuropsychological and clinical data

All the questionnaires and tests that will be used in this study are listed below. The scores they yield will allow us to identify determinants (social interactions, social abilities, cognitive skills, and personality traits) that could explain the potentially beneficial impact of the different social contexts on participants’ learning. These will then be used to define explanatory variables for differences in memory performance depending on social learning context.

#### Social interactions and social cognition

Three questionnaires will be used to identify the patients’ social interaction skills, in order to find out which social variables could explain differences in performance between the collaboration or observation context and the presence-only context. These questionnaires assess the quality of people’s social relationships [56], changes in socio-emotional [57] and exo-/egocentric behaviour since the onset of the disease, and social vulnerability [58].

Social cognition skills will be assessed with an empathy questionnaire [59, 60], and three tests developed in our research unit: a test of knowledge of social rules [61], a preference judgment test adapted from [62], and a social learning information test.

#### Memory abilities

This protocol will include several tests used in clinical neuropsychology to determine memory abilities. More specifically, we will assess visual episodic memory [63], working memory [64], and semantic memory [65, 66].

#### Personality traits

Personality traits will be assessed with the Temperament and Character Inventory developed by Cloninger [67]. This inventory is based on two fundamental components: temperament, which qualitatively describes the psychobiological dimensions of the individual’s personality; and character, which describes the individual’s levels of adaptation and maturity. The two subscales that are most relevant for the purposes of the study concern the character component. They are *Self-Directedness*, which corresponds to the level of individual maturity, and *Cooperativeness*, which reflects social maturity.

#### Additional assessment

Global cognitive impairment will be assessed using the MoCA [51]. Finally, executive functions in everyday life will be assessed with a questionnaire [68]. An MRI scan may be carried out to document patients’ brain lesions.

#### Demographic data

The relationship with the dyadic partner will be documented: nature of relationship (relative, friend, other), feeling of closeness, and number of years they have known each other. Some demographic information will also be collected: age, sex, highest degree obtained, living alone or not.

### MEM&SO study procedure

MEM&SO is a comparative cross-sectional study. Participants will be recruited from several centres (Caen, Rennes, Rouen, and memory clinics in Normandy). HC will be recruited in and around Caen, and the examinations of all participants will be performed in Caen. Registered on ClinicalTrials.gov (no. NCT05800028).

**Fig. 3:**
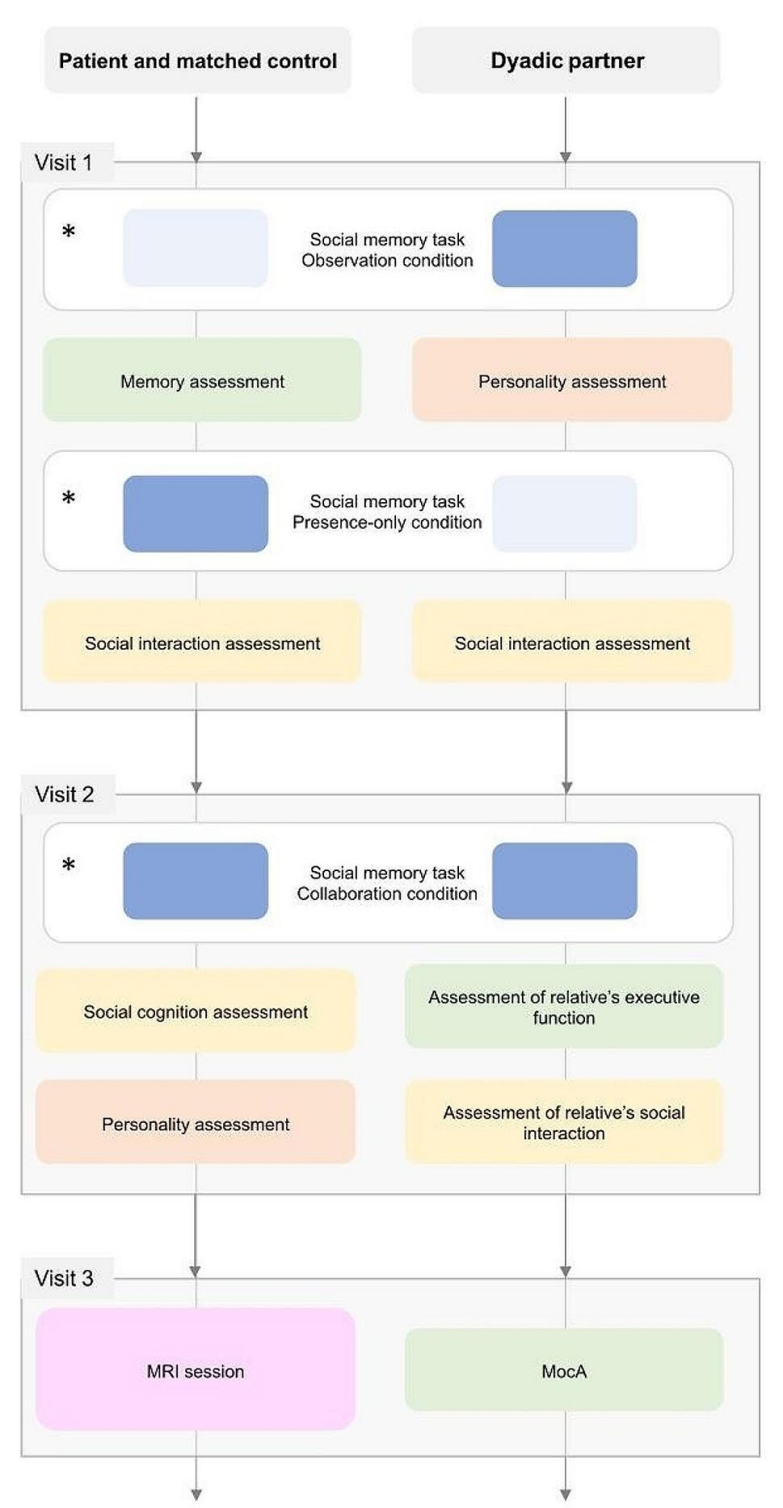
Schedule for assessment visits. *The order of the three contexts will be randomly counterbalanced across the dyads

After the inclusion phase, the study will take place over 3 half-days, corresponding to the three study visits (see Fig. 3). Each visit will last 90 min, with a 10-minute break in the middle.

### Data analysis plan

Each participant’s performance in the presence-only context will serve as a reference score, and a differential score will be calculated by subtracting this reference score from the participant’s memory performance in the collaboration context. The *z* score of this differential score will then be calculated, based on the mean and standard deviation for the HC group. This will show whether each patient’s differential score differs significantly from that of the HC group. The same analyses will then be carried out for performance in the observation context.

As this study is designed to identify determinants (social interactions and abilities, cognitive skills, and personality traits) that could explain the differential scores for the collaboration and observation contexts, we will carry out multiple comparative case analyses [48–50], based on the differential scores for the collaboration context of at least three patients whose memory performance is substantially better in the collaboration condition, compared with the presence only condition, and for three patients whose performance is only minimally better. These six patients will be compared on their social skills (social interactions and abilities), personality (especially the cooperation and determination dimensions), and memory performance in classic neuropsychological tests. Following a logic of replication, other patients will be added to the analyses, to see whether a regular pattern emerges. The same analyses will be conducted on differential scores for the observation context.

## Discussion

Given that individuals with neurodegenerative diseases may need to learn new information despite their cognitive disorders, establishing whether social interaction favours this learning could lead to the development of new cognitive rehabilitation techniques. The purpose of the present study will therefore be to find out whether a particular social context can improve the memory performance of patients with neurodegenerative diseases, and shed light on cognitive, social and personality variables that could subtend differences in memory performance between social learning contexts (i.e., collaboration or observation vs. presence-only).

As this is a multiple comparative case study, cognitive, social and personality profiles will be established for each target population, in order to identify the most beneficial learning context for each patient. Depending on the patient’s profile, strategies can then be devised to compensate for cognitive deficits.

The MEM&SO protocol will therefore have both theoretical and practical implications in the field of social cognition, bringing social psychology concepts to neuropsychology.

## Data Availability

Not applicable.

## Declarations

### Trial status

The recruitment phase began in June 2023. To date, only 5 dyads (10 participants) have been included in the study (April 2024).

## Abbreviations

MEM&SO: Memory and social interactions in neurodegenerative disease
AD: Alzheimer disease
svPPA: Semantic variant of primary progressive aphasia
ToM: Theory of mind
HC: Healthy control
MoCA: Montreal cognitive assessment
